# Effects of large-scale Amazon forest degradation on climate and air quality through fluxes of carbon dioxide, water, energy, mineral dust and isoprene

**DOI:** 10.1098/rstb.2007.0027

**Published:** 2008-02-11

**Authors:** Richard Betts, Michael Sanderson, Stephanie Woodward

**Affiliations:** Met Office Hadley CentreExeter EX1 3PB, UK

**Keywords:** climate change, deforestation, Amazon dieback, feedbacks, carbon cycle, biogeochemical cycles

## Abstract

Loss of large areas of Amazonian forest, through either direct human impact or climate change, could exert a number of influences on the regional and global climates. In the Met Office Hadley Centre coupled climate–carbon cycle model, a severe drying of this region initiates forest loss that exerts a number of feedbacks on global and regional climates, which magnify the drying and the forest degradation. This paper provides an overview of the multiple feedback process in the Hadley Centre model and discusses the implications of the results for the case of direct human-induced deforestation. It also examines additional potential effects of forest loss through changes in the emissions of mineral dust and biogenic volatile organic compounds. The implications of ecosystem–climate feedbacks for climate change mitigation and adaptation policies are also discussed.

## 1. Introduction

The climate and air quality in Amazonia depend strongly on the character of the vegetation cover, through its influence on the physical properties of the land surface properties and biogeochemical fluxes. Large-scale changes in vegetation cover, for example a reduction in the current forest area, would therefore be expected to modify the local climate. Moreover, a reduction in forest cover would also be expected to contribute to global climate change through the release of stored carbon contributing to the rise in atmospheric CO_2_.

Vegetation cover change, mostly in the form of deforestation, is currently occurring as a direct result of human activities in the Amazon region. By 2001, the original forest area of approximately 6.2 million km^2^ had been reduced to 5.4 million km^2^, 87% of the original area (Malhi *et al.* 2008). Current plans for infrastructure expansion and integration could further reduce forest cover to 3.2 million km^2^, which is 53% of the original area, by 2050 (Soares *et al.* 2006).

Global climate change may also lead to changes in the Amazonian vegetation cover, especially if it leads to significant reductions in precipitation in this region. The relationship between the warming of global average temperatures and changes in regional precipitation patterns is highly uncertain, but a number of climate models suggest that global warming could lead to particular patterns of warming in the north Atlantic and tropical east Pacific sea surface temperatures (SSTs), which change the atmospheric circulation such that precipitation is reduced across part or all of Amazonia. (Good *et al.* 2008; Harris *et al.* 2008). Strong drying of Amazonia or northeast South America is simulated by variants of the Hadley Centre climate model (Betts *et al.* 1997; Cox *et al.* 2000; Murphy *et al.* 2004), although it must be emphasized that many other climate models do not simulate such a drying in this region (IPCC 2007; Li *et al.* 2008).

This paper reviews simulations performed with the Hadley Centre climate model including changes in the vegetation cover to quantify and compare several processes through which large-scale Amazon forest degradation may affect climate. Specifically, these involve changes in the physical properties of the land surface (Betts *et al.* 2004), and net emissions of carbon dioxide, dust and isoprene to the atmosphere (Cox *et al.* 2000; Sanderson *et al.* 2003; Woodward *et al.* 2005). The discussion considers the roles of these effects as feedbacks on global climate change, should this lead to a drier climate and forest loss in Amazonia and also their roles as forcings of climate change due to direct human-induced deforestation.

## 2. Biophysical effects of forest degradation on regional and global climates

In the HadCM3LC coupled climate–carbon cycle model, the regional warming and drying of the Amazonian climate simulated for the twenty-first century lead to a ‘dieback’ of large areas of forest (figure 1*a*). The forest loss itself plays a key role in the simulated drying of the Amazonian climate. Relative to bare soil, vegetation (especially forest) can enhance the evaporative flux of moisture to the atmosphere through the extraction of moisture deep in the soil by plant roots for transpiration. Furthermore, the vegetation canopy can capture a greater fraction of precipitation that is then re-evaporated back to the atmosphere, compared with bare soil that holds less water on the surface before run-off and infiltration. In addition, the higher aerodynamic roughness of a vegetated land surface can promote the flux of moisture to the atmosphere through enhanced turbulence. Changes in the nature of vegetation cover, particularly from forest to non-forest, can therefore significantly alter the surface moisture budget and exert further effects on the surface energy budget. Forest loss reduces evaporation, causing a greater proportion of the available energy at the land surface to flow to the atmosphere in the form of sensible heat rather than latent heat; this exerts a warming influence on the near-surface air temperature. Reduced evaporation also reduces the flux of moisture to the atmosphere, potentially decreasing the quantity of moisture available for precipitation.

Betts *et al.* (2004) examined these feedbacks with two simulations with HadCM3LC, one including interactive vegetation and the other with global vegetation cover fixed at the present-day state. In order to remove carbon cycle feedbacks and isolate the biogeophysical feedbacks, CO_2_ concentrations were prescribed to the standard IS92a scenario in both simulations. This scenario projects the atmospheric CO_2_ concentration to rise to 713 ppmv by 2100. For comparison, pre-industrial and present-day concentrations are 278 and 378 ppmv, respectively.

The general global patterns of climate change were similar in the two simulations, with almost all changes in temperature and precipitation being of the same sign irrespective of the inclusion of vegetation feedbacks. This implies that vegetation feedbacks do not have significant influence on the atmospheric circulation in comparison with the greenhouse gas (GHG) forcing. However, some of the regional climate changes were significantly affected by vegetation feedbacks. In particular, the precipitation reduction over Amazonia was greater with interactive vegetation than with prescribed present-day vegetation. With present-day vegetation, the precipitation reduced by 1.9 mm d^−1^, but with forest dieback the reduction was −2.4 mm d^−1^ (table 1). Biogeophysical feedbacks from the forest dieback therefore enhanced the local drying by approximately 26%. In the western part of the basin, the feedback was greater still, magnifying the precipitation reduction by over 30% (figure 1*b*). The larger precipitation decrease in western Amazonia was attributed to drought-induced dieback of the eastern forests contributing to further rainfall reductions in the west. The forest loss also increased surface albedo that reduced convection and moisture convergence, providing a further positive feedback on rainfall reduction. The Amazon forest dieback therefore magnified the local drying of the climate, providing a reason for why drying in this model is more extreme than the other climate models.

This result is consistent with the previous model results, suggesting that human-induced deforestation would impact the regional climate of Amazonia, principally by reducing local precipitation and increasing temperature (Lean & Rowntree 1997). It therefore provides further evidence that forest degradation, by whatever cause, would lead to a hotter, drier climate in Amazonia.

The positioning of Amazonia on the equator means that large-scale forest loss could also exert more far-reaching effects by modifying the global atmospheric circulation. Amazonia lies beneath the region of ascending air that moves northwards and southwards across the equator with the seasons, and which derives its energy from the solar heating of the Earth's surface below. With forest present, higher rates of evaporation cause a larger proportion of the energy to be transferred to the atmosphere in the form of latent heat, which allows energy to be transported higher into the atmosphere before conversion to sensible heat upon condensation of the water vapour. This mechanism drives deep convection that enhances ascent and the overturning motion of the Hadley circulation. Gedney & Valdes (2000) found that, in a climate model, removal of the Amazonian forest caused more energy to be transferred to the atmosphere as sensible heat, heating the lower atmosphere rather than higher levels and providing a weaker driver of ascent. The subsequent reduction in the Hadley circulation modified the atmospheric circulation at higher latitudes through the poleward propagation of Rossby waves, altering regional climates many thousands of kilometres from Amazonia.

## 3. Contribution of forest degradation to rising CO_2_ and global warming

The forest dieback in HadCM3LC also exerted feedbacks on global and local climate changes through the carbon cycle, and again these were isolated by further HadCM3LC simulations in which various processes were enabled or disabled (Betts *et al.* 2004; Cox *et al.* 2004).

In a HadCM3LC simulation that simulated carbon fluxes between the atmosphere, oceans and terrestrial biosphere, but in which the radiative forcing by rising CO_2_ was omitted, uptake of carbon by the oceans and terrestrial biosphere due to increased dissolution in ocean waters and enhanced photosynthesis caused the rise in CO_2_ to be approximately half the rate of anthropogenic emissions throughout both the twentieth and twenty-first centuries (table 2). The simulated CO_2_ concentration at 2100 was 700 ppmv (table 2), close to that in the standard IS92a scenario provided to the prescribed CO_2_ simulations described in §2, which similarly was generated without consideration of the effects of climate change on the carbon cycle. Relative to pre-industrial, the total uptake of carbon by the global oceans by 2100 was 367 gigatonnes of carbon (GtC), and uptake by the terrestrial biosphere was 633 GtC by 2100, with 64 GtC of this being in South American vegetation (largely in Amazonia).

However, in the simulation with CO_2_ radiative forcing included, the climate change led to a number of changes in the oceanic and terrestrial carbon cycles that overall exerted a positive feedback on the CO_2_ rise and global warming (table 1). Ocean carbon uptake increased to 495 GtC by 2100, but this was more than offset by the terrestrial biosphere becoming an overall net source of carbon instead of a sink. The main process was an increase in soil respiration in response to higher temperatures, but Amazonian forest dieback also played a part. The overall loss of carbon from the terrestrial biosphere relative to pre-industrial was 98 GtC, with global soils losing approximately 150 GtC and South American vegetation losing 73 GtC—vegetation elsewhere in the world still largely gained carbon—and the total global vegetation carbon uptake was approximately 60 GtC. Compared with the uptakes when climate change was excluded, the global terrestrial carbon deficit was therefore 731 GtC, with 137 GtC of the deficit coming from Amazonian vegetation carbon decreasing rather than increasing. The overall atmospheric increase, accounting for both ocean and terrestrial feedbacks, was 590 GtC, so Amazonian forest dieback provided 22% of this global feedback.

In the simulation with CO_2_ concentrations prescribed to the IS92a scenario that ignored climate–carbon cycle feedbacks, global average temperature rose by 4°C (table 1). When carbon cycle feedbacks were included, global warming was 5.5°C (table 1). Approximating the global temperature response to be proportional to the CO_2_ rise, the Amazon forest dieback therefore increased global warming by approximately 0.3°C. Compared with the non-feedback warming of 4°C, Amazon forest loss increased the rate of twenty-first century global warming by approximately 8%.

The regional drying in Amazonia was also more severe in the simulation with carbon cycle feedbacks than that with these feedbacks neglected. Without carbon cycle feedbacks the precipitation reduction had been −2.4 mm d^−1^, but with carbon cycle feedbacks the reduction was −3.0 mm d^−1^. Assuming the local precipitation change to be linearly related to global mean temperature change, Amazon forest dieback therefore further enhanced the local drying by approximately 0.05 mm d^−1^ through its contribution to global carbon cycle feedbacks.

As a feedback on global warming, the process of Amazon forest loss relies on particular responses of the regional climate to the radiative forcing. Since not all models produce a drying climate in Amazonia under global warming, this component of the carbon cycle feedback is highly uncertain. However, the results presented here also help to quantify the potential contribution of direct forest degradation as a driver of climate change (as opposed to a feedback). If this area of forest were lost through direct human action instead of as a result of climate change, the 73 GtC released from the vegetation would be a direct contribution to the CO_2_ rise.

## 4. Multiple feedbacks between climate change and forest degradation

The Amazonian drying and forest dieback in HadCM3LC is therefore a complex coupled process involving multiple interactions between atmospheric CO_2_, radiatively forced climate change, regional temperature and precipitation patterns, and vegetation. The drying is initiated by atmospheric circulation responses to particular patterns of SST change, associated mainly with radiatively forced climate change but also modified by physiological forcing of climate via vegetation responses (Betts *et al.* 2004). Despite CO_2_ fertilization, the climate warming and drying cause forest dieback that then exerts two positive feedbacks on the precipitation reduction: reduced forest cover causes further suppression of local evaporative water recycling (a biogeophysical feedback); and carbon release contributes to a global positive feedback on CO_2_ rise, which accelerates global warming and magnifies the associated patterns of precipitation change (figure 2).

This analysis helps to explain why the Amazonian precipitation reduction simulated by HadCM3LC is more extreme than that simulated in other GCMs. In the fully coupled climate–carbon cycle simulation, approximately one-third of the precipitation reduction in Amazonia is attributable to a combination of biogeophysical and global carbon cycle feedbacks. In addition, a small part of the precipitation reduction is attributable to physiological forcing by the rise in CO_2_ concentration, both in Amazonia and across the globe. These processes are often not included in other GCM simulations of future climate change.

Direct human-induced forest degradation could initiate parts of the above multiple feedback process by emitting CO_2_ and reducing evaporation (figure 2).

## 5. Increased dust production and its effects on radiative forcing

Forest degradation could result in increased exposure of bare soil, especially if this were accompanied by a drying climate. This raises the possibility of further effects on climate through the release of mineral dust—this can affect climate by exerting radiative forcings in both the short wave and the long wave. The net effect is complex and depends on other factors such as the albedo of the underlying surface.

Woodward *et al.* (2005) used a fully interactive dust scheme (Woodward 2001) within the atmospheric component of HadCM3LC to simulate the changes in atmospheric dust load as a consequence of global vegetation change including forest loss and the associated drying climate in Amazonia. The model included six size classes of dust from 0.03 to 30 μm radius, and produced dust from the bare soil fraction of a grid box when the friction velocity exceeds a threshold, which depends on soil moisture and particle size. Horizontal and vertical dust flux calculations are based on those of Marticorena *et al.* (1997). Dry deposition through gravitational settling and turbulent mixing in the boundary layer and below cloud scavenging processes are included. Radiative properties were calculated using refractive index data from a range of sources, in an attempt to produce globally representative values, rather than properties applicable to one particular source region.

Two 10-year simulations were performed with the atmospheric model HadAM3, using prescribed vegetation states, SSTs and CO_2_ concentration obtained from the simulations for 2000 and 2100 in the HadCM3LC coupled climate-carbon cycle simulation described in §2. In the 2100 simulation, Amazonia was a greater dust source than the present-day Sahara (figure 3) due in part to loss of vegetation cover and drying of the soil. However, the area of bare soil was much smaller than the Sahara and the soil was not as dry. The strength of the new dust source in Amazonia was largely due to increased speed of the surface winds, which occurred as a consequence of reduced aerodynamic roughness of the landscape due to loss of the forest. This reflects the fact that dust flux increases with the cube of the wind speed, but only linearly with area.

Mineral dust absorbs and scatters incoming short-wave radiation, giving a negative surface forcing, but the change in top of the atmosphere short-wave flux depends not only on the properties of the dust, such as size distribution and refractive index, but also on the underlying albedo. Short-wave top of the atmosphere forcing tends to be positive over bright surfaces such as ice and deserts or over cloud, and negative over dark surfaces such as ocean or forests.

Dust absorbs long-wave radiation, and the top of the atmosphere long-wave forcing is positive. In the case of the Amazonian dust over the source region, the long-wave forcing dominates, but the short-wave forcing is also predominantly positive, leading to decadal mean positive net forcing in excess of 10 W m^−2^ locally (figure 3). The equivalent net surface forcing is negative and also exceeds 10 W m^−2^.

The experiments were designed to calculate the direct radiative forcing due to dust excluding any feedbacks, and as such do not simulate changes in climate due to the dust. However, it may be supposed that the cooling of the surface and the warming aloft caused by the dust would tend to reduce convection and low-level winds, thus producing a negative feedback on dust production. Lower surface temperatures could also result in reduced evaporation and a somewhat moister soil, again producing a negative feedback. However, these effects are likely to be much smaller than the climate changes driving the desertification of Amazonia.

The dust produced by the drier, windier, desertified Amazonia was transported considerably beyond the confines of the Amazonian region itself (figure 3). Particularly high atmospheric dust loads were simulated above the equatorial east pacific, but dust loads were also increased above the whole tropical Pacific. Dust loads also increased over the north and south Atlantic, although it is difficult to determine the relative contributions of the Amazonian and Saharan dust sources to these. SST anomalies in the equatorial east Pacific and the Atlantic, and in particular the north–south SST gradient in the Atlantic, have been identified as drivers of regional climate change in Amazonia (Cox *et al.* 2004; Good *et al.* 2008; Harris *et al.* 2008), and these SSTs could be affected by the radiative forcing exerted by changes in dust loading above (figure 3). Emissions of mineral dust aerosol from Amazonia could therefore provide a further feedback on the regional climate change by modifying the SSTs and the associated atmospheric circulations.

These results also have important implications regarding the effects of human-induced forest degradation. Although the drying of the Amazonian climate due to global warming is uncertain, large-scale removal of the forest could expose more bare soil and also lead to local precipitation reductions as discussed in §2. This could lead to increased dust emissions. Moreover, increased wind speed due to forest loss has been identified as a key factor in increasing dust emissions. These results suggest that Amazonia has the potential to become a significant new dust source, whether forest degradation occurs through global warming or direct human action.

## 6. Changing emissions of biogenic volatile organic compounds and their effects on radiative forcing and air quality

Changes in the cover of vegetation exert a further impact on climate, radiative forcing and air quality, via surface ozone levels. Tropospheric ozone levels have increased since the pre-industrial era (Volz & Kley 1988), which exert a positive radiative forcing (IPCC 2007). Vegetation emits a wide range of volatile organic compounds (referred to as BVOCs), of which the most important is isoprene. These BVOCs are highly reactive with correspondingly short lifetimes (Kesselmeier & Staudt 1999). They can create or destroy ozone, depending on the levels of nitrogen oxides (NO_*x*_,=[NO+NO_2_]). When NO_*x*_ levels are low, these BVOCs will react directly with ozone, reducing its levels. However, when NO_*x*_ levels are larger, net ozone production occurs. The consequences of forest degradation in the Amazon region would be to reduce the emission of BVOCs, with a subsequent impact on ozone levels at the surface.

The impact of Amazon forest degradation and other global vegetation changes on BVOC emissions and surface ozone levels was studied by Sanderson *et al.* (2003). These authors used the HadCM3LC model coupled to a global Lagrangian chemistry model, STOCHEM (Collins *et al.* 1997). The emissions of the BVOCs were calculated using the algorithms developed by Guenther *et al.* (1995), which use temperature, radiation intensity and various plant data, such as leaf area index and vegetation type. For this study, the vegetation changes were calculated using the prescribed levels of CO_2_. The direct effect of CO_2_ on isoprene was not included (Rosenstiel *et al.* 2003). There was no direct feedback between climate and changes in carbon uptake or loss by the vegetation, but the vegetation can change dynamically in response to the changes in climate. Isoprene emissions and surface ozone levels were simulated for the 1990s and 2090s. Two simulations were performed for the 2090s, one with 1990s vegetation and the other with 2090s vegetation including Amazon forest dieback, so the impact of changed vegetation on projected future ozone levels could be assessed. Global emission totals of anthropogenic pollutants were taken from the IS92a scenario and distributed over the globe according to the IPCC SRES A2 scenario.

With the vegetation distribution fixed at that for the 1990s, isoprene emissions were projected to increase from 550 to approximately 740 Tg yr^−1^ by 2100. However, a smaller increase to 700 Tg yr^−1^ was simulated when the 2090s vegetation distribution was used. Isoprene emissions were therefore approximately 40 Tg yr^−1^ lesser if vegetation change was included in the simulations.

Changes in summertime surface ozone levels between the 2090s and the 1990s are shown in figure 4. When the vegetation distribution was fixed at the 1990s state (figure 4*a*), ozone levels over Amazonia were projected to be up to 25 ppbv larger in the west and 5–15 ppbv larger in the east. When the changed vegetation distribution is used (and global isoprene emissions are smaller), the increase in surface ozone levels is projected to be approximately 5 ppbv smaller in eastern Amazonia (figure 4*b*).

A significant loss mechanism for ozone is dry deposition, where ozone is irreversibly removed at the surface. Deposition to vegetation is the major loss route, thus any changes in vegetation will also affect the dry deposition sink. However, the global deposition fluxes calculated for the two future simulations were almost identical and differed by less than 1%. The simulations for the 2090s included the effect of increasing levels of CO_2_ on dry deposition via reduced stomatal conductance (Sanderson *et al.* 2007). Reduced stomatal opening due to higher CO_2_ led to a reduced flux of ozone into the stomatal cavities within leaves. For these particular simulations, the increase in deposition fluxes caused by larger surface ozone values has been at least partly offset by reduced stomatal conductance.

Changes in the dry deposition sink are therefore not the cause of the different future ozone levels in these simulations.

Isoprene emissions have a significant impact on the projected future surface ozone levels. Ignoring vegetation changes has meant that future simulated ozone levels were greater by 5–10 ppbv, owing to larger isoprene emissions. This may have implications for air quality in the region, with potential implications for the health of humans, animals, ecosystems and crops. Although tropospheric ozone is a GHG, so a relative reduction in ozone due to Amazon forest dieback would provide a negative feedback on radiatively forced climate change, the changes simulated here as a consequence of Amazon forest degradation would exert only a minor radiative forcing so that they are likely to provide only a small feedback on climate change. However, this feedback effect could be larger if the ozone changes affected carbon uptake by vegetation with consequent effects on atmospheric CO_2_ (Sitch *et al.* 2007). A reduction in surface ozone concentrations would decrease the damaging effect of ozone on plants and therefore partly ameliorate any reduction in carbon uptake that may occur as a result of ozone poisoning. Carbon uptake could therefore be slightly larger as a consequence of the reduced isoprene emissions, providing a further negative feedback on climate change.

## 7. Conclusions

It is concluded that future forest degradation in Amazonia could interact with climate and air quality in complex ways, acting as both a feedback on climate change from other causes and a driver of climate change in its own right. The extreme twenty-first century precipitation decrease and forest dieback simulated in Amazonia by the HadCM3LC coupled climate–carbon cycle model are a coupled process emerging from multiple interactions between the atmosphere, the oceans and the land ecosystems of the Amazon and elsewhere. Following Amazon forest degradation, by whatever cause, biogeophysical and carbon cycle effects can all act to reduce the local precipitation, although it can be speculated that dust effects may partially decrease drying. Isoprene emissions, affecting local air quality through ozone concentrations, may also be affected by forest degradation. Global emission reductions policies may need to take account of these feedbacks and their associated uncertainties if GHG stabilization targets are to be aimed for. Policies to avoid deforestation in Amazonia may have greater benefits than previously assumed, through both reducing the vulnerability of wider areas of forests and facilitating easier adaptation to climate change.

## Figures and Tables

**Figure 1:**
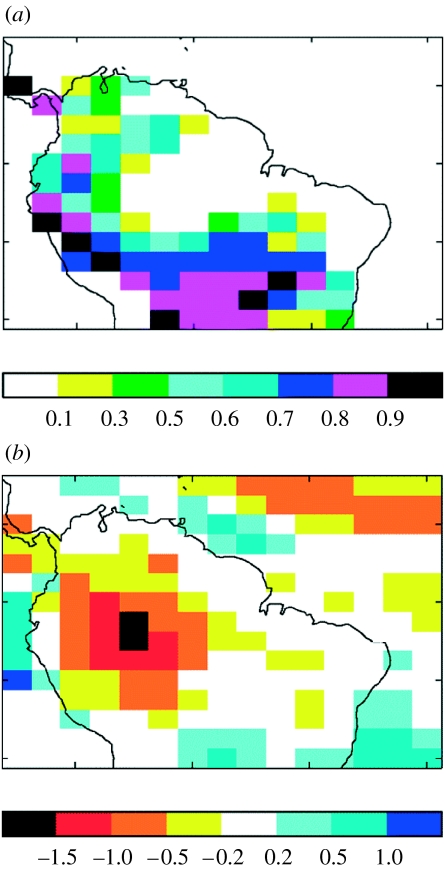
(*a*) Fractional coverage of the ‘broadleaf tree’ plant functional type simulated for decades throughout the twenty-first century by the HadCM3LC coupled climate-carbon cycle model driven by the IS92a emissions scenario. (*b*) Difference in precipitation (mm d^−1^) between simulations with forest loss as shown in (*a*) with vegetation fixed at pre-industrial state (30-year mean centred approx. 2080).

**Figure 2:**
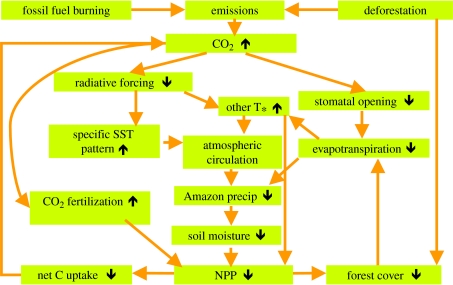
Schematic of potential feedback processes involved in Amazonian climate change and forest degradation, involving either or both global warming or direct human impacts on the forest. Feedbacks involving specific SST changes, atmospheric circulation and Amazon precipitation rely on particular responses of regional climate change in the Hadley Centre climate models—these are seen in some other models, but not all. A large number of studies suggest impacts on regional climate through reduced evapotranspiration following deforestation. T_*_, surface temperature.

**Figure 3:**
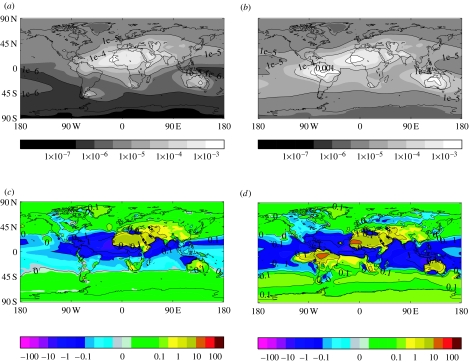
Effects of climate change and associated vegetation responses on atmospheric dust load and radiative forcing, simulated with the HadAM3 climate model. This includes a drying of the Amazonian climate and loss of the Amazon forest. (*a*) Atmospheric dust load (k g m^−2^) at 2000, (*b*) atmospheric dust load (kg m^−2^) at 2100, including changes in climate and vegetation change from HadCM3LC, (*c*) net top of atmosphere radiative forcing due to dust at 2000 and (*d*) similar to (*c*) but for 2100.

**Figure 4:**
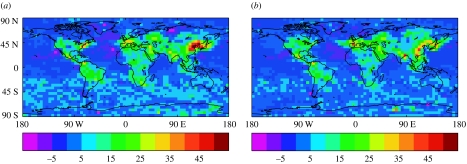
Effects of climate change and associated vegetation responses on summertime (June, July and August) mean surface ozone levels (ppbv): changes between the 1990s and the 2090s. (*a*) Vegetation distribution fixed at 1990s state, (*b*) vegetation fixed at state simulated for 2090s due to climate change in HadCM3LC. A positive value indicates that ozone levels are greater in the 2090s than the 1990s.

**Table 1:** Changes in global temperature and Amazonian precipitation in simulations with and without biogeophysical and carbon cycle feedbacks.

	prescribed CO_2_ rise, fixed vegetation	prescribed CO_2_ rise, interactive vegetation exerting biophysical feedbacks	fully interactive carbon cycle
atmospheric CO_2_ at 2080s	713	713	980
global mean temperature change by 2080s relative to pre-industrial era (°C)	4.0	4.0	5.5
Amazonian precipitation change by 2080s relative to pre-industrial era (mm d^−1^)	−1.9	−2.4	−3.0

**Table 2:** Global and South American carbon storage changes between 1860 and 2100 with and without effects of climate change on the carbon cycle (adapted from Cox *et al.* 2004).

	without climate effects on the carbon cycle	with climate effects on the carbon cycle	change due to climate change
anthropogenic CO_2_ emissions (GtC)	1883	1883	not considered
South American vegetation carbon change (GtC)	64	−73	−137
South American soil carbon change (GtC)	76	−55	−131
total South American land carbon change (GtC)	140	−128	−268
global total land carbon change (GtC)	633	−98	−731
global ocean carbon change (GtC)	367	495	128
atmospheric carbon change (GtC)	883	1486	603
atmospheric CO_2_ at 2100 (ppmv)	700	980	280
